# Engineering of optogenetic devices for biomedical applications in mammalian synthetic biology

**DOI:** 10.1049/enb2.12022

**Published:** 2022-07-07

**Authors:** Ningzi Guan, Xianyun Gao, Haifeng Ye

**Affiliations:** ^1^ Synthetic Biology and Biomedical Engineering Laboratory Biomedical Synthetic Biology Research Center Shanghai Key Laboratory of Regulatory Biology Institute of Biomedical Sciences and School of Life Sciences East China Normal University Shanghai China

## Abstract

Gene‐ and cell‐based therapies are the next frontiers in the field of medicine. Both are transformative and innovative therapies; however, a lack of safety data limits the translation of such promising technologies to the clinic. Improving the safety and promoting the clinical translation of these therapies can be achieved by tightly regulating the release and delivery of therapeutic outputs. In recent years, the rapid development of optogenetic technology has provided opportunities to develop precision‐controlled gene‐ and cell‐based therapies, in which light is introduced to precisely and spatiotemporally manipulate the behaviour of genes and cells. This review focuses on the development of optogenetic tools and their applications in biomedicine, including photoactivated genome engineering and phototherapy for diabetes and tumours. The prospects and challenges of optogenetic tools for future clinical applications are also discussed.

## 1 INTRODUCTION

Optogenetic technology combines optics and genetics to control cell activities in a non‐invasive manner using light. Optogenetics was initiated in 2005, when blue light was first used to control electrical changes in nerve cells with millisecond precision [1]. One year later, an optogenetic tool was used to restore visual responses in animals [2]. By 2010, optogenetics was rated as one of the major breakthroughs of the preceding decade by *Science* and the method of the year by *Nature Methods* [3].

In recent years, optogenetics has rapidly developed as a non‐invasive technique with high spatiotemporal specificity, reversibility, and few toxic side effects [4]. Dozens of optogenetic tools have been developed using synthetic biology methods to control gene expression [5], genome editing [6], cell signalling pathways [5], cell migration, and organelle motion [7]. These tools have been applied in the development of control systems for biomedicine as phototherapy for metabolic diseases [8], cardiovascular diseases [9], neurological diseases [10], tumours [11], and various other diseases. Optogenetics has started a new era in gene‐ and cell‐based therapies. In this review, we introduce recent advances in optogenetic tools, in terms of their development, followed by potential applications in the medical domain. First, the application of optogenetic tools to genome engineering, including gene recombination, gene editing, and gene transcriptional regulation is summarised owing to their key roles in basic scientific research, as well as disease treatment. Further, potential optogenetic‐controlled gene‐ and cell‐based therapies for diabetes and tumours are introduced based on their impact on health and the urgent need for precise therapies to replace inefficient traditional treatments. To promote future improvements in optomedicine, defects in the current optogenetic circuits are also discussed and prospective strategies for the development of novel optogenetic tools are proposed.

### 1.1 Development of optogenetic tools

Optogenetics, a combination of optics and genetics, can precisely control the spatial and temporal activities of specific cells through the assembly of photosensitive elements and regulatory modules. As basic components of optogenetic tools, optocontrolled proteins can detect and respond to specific light wavelengths. Optocontrolled proteins originate from a wide range of sources, including bacteria, fungi, algae, plants, and animals [12]. They can be subdivided into several categories based on principles of light response as follows: photoreceptors based on a photoactivated cation channel [13, 14], photosensitive proteins associated with allosteric regulation induced by light [11, 15], photosensitive proteins that can undergo photoinduced oligomerisation or dissociation [16, 17], and photosensitive proteins that are responsive to photolysis [18]. Here, we selectively introduce several photosensitive proteins that have been widely used to construct optogenetic tools for biomedical engineering.

The photosensitive protein UVR8 (UV response locus 8) is a component of the UVB (ultraviolet B; 285–315 nm) response signalling pathway in *Arabidopsis thaliana* [19]. It absorbs UVB via a chromophore formed by the tryptophan residues in the absence of any cofactors. UVR8 is a homodimer in the dark and dissociates and binds to the COP1 protein under UV light stimulation to form a heterodimer [20]. Photocleavable protein (PhoCl) is a violet‐light‐responsive fluorescent protein. A *β*‐elimination reaction induced by violet light (∼400 nm) results in photocleavage of the polypeptide backbone to form a small peptide and large ‘empty barrel’ fragment that spontaneously dissociates [21]. It has been used to engineer light‐activatable Cre recombinase, the Gal4 transcription factor, and viral protease. However, both the optocontrolled dimerisation of UVR8–COP1 and the photocleavage reaction of PhoCl are irreversible. Additionally, the strong phototoxicity of UVB and violet light limits their biomedical application.

Blue light‐responsive proteins are the most commonly used photosensors for the construction of optogenetic tools. Channelrhodopsin‐2 (ChR2) from *Chlamydomonas reinhardtii* was the earliest photosensitive protein used in the field of neuronal optogenetics [1]. It is a light‐responsive ion channel that is activated by blue light and leads to the influx of Na^+^, Ca^2+^, and K^+^ [22]. However, the relatively slow kinetic properties of ChR2, the most widely studied opsin, limit its use in neuroscience. Chronos is a blue and green light‐driven channel rhodopsin with faster kinetics and high light sensitivity [23]. It enables high‐frequency neural activation and has great potential for the manipulation of auditory pathways in optogenetic‐based neuroprostheses [24]. Another group of opsins is the light‐responsive G‐protein‐coupled receptors, such as melanopsin, which triggers a phosphodiesterase‐dependent cascade, resulting in a calcium response in the intrinsic photosensitive retinal ganglion cells that correlates with the action potential induced upon blue light illumination. Specifically, blue light induces photoisomerisation of the 11‐cis retinal chromophore, changes the conformation of melanopsin, and then sequentially activates the Gaq‐type G protein, phospholipase C, and phosphokinase C. Subsequently, transient receptor potential channels (TRPCs) are opened, resulting in depolarisation of the cell membrane and an influx of Ca^2+^ [13].

The light‐oxygen‐voltage domain (LOV) is a caging domain isolated from the plant photoreceptor phototropin 1 and is involved in plant growth regulation by responding to blue light [25]. It exists widely in plants, algae, bacteria, and fungi [26, 27]. The AsLOV2 (*Avena sativa* LOV2) domain is considered an example of this domain, and it is most commonly used to construct optogenetic devices [28], in which an effector domain is fused to the C‐terminal Jα helix of LOV2 and caged via steric hindrance in the dark [29]. Blue light induces conformational changes in AsLOV2, thereby resulting in the unfolding of the Jα helix and the exposure of effectors to restore their biological functions [30]. Notably, a light‐induced protein dissociation system (LOVTRAP) composed of AsLOV and ZDK proteins was constructed [31], in which ZDK dissociates from the LOV domain upon blue light irradiation. Vivid (VVD) is the smallest LOV domain‐containing protein. Blue light triggers conformational changes in the N‐terminal cap (Ncap) of the VVD PAS domain, leading to the rapid exchange of VVD dimers [32]. To address the limitations of VVD, including low efficiency and selectivity due to homodimerisation, low switch‐off kinetics, and low dimerisation affinity, VVD variants were developed as pairs of distinct photoswitches named Magnets. The interaction between pMag and nMag proteins induced by blue light is based on electrostatic interactions, which prevent unwanted homodimerisation [33]. Additionally, several photoreceptors, such as EL222 and FKF/GI, are light‐controlled dimerisation systems based on the LOV domains [34, 35].

Cryptochrome 2 (CRY2) is a blue light‐absorbing photosensor found in plants and animals with an N‐terminal photolyase homology region (PHR) that binds flavin and pterin chromophores and mediates light responsiveness [36]. It was found to form oligomeric clusters in response to blue light in mammalian cells and return to a diffuse initial state within minutes upon the withdrawal of blue light [7]. However, the homologous oligomerisation of wild‐type CRY2 requires high local protein concentrations or multivalent protein partners. Therefore, an improved CRY2olig module containing the E490G mutation was developed for rapid, reversible, and robust clustering (within seconds) in response to light [37]. In addition, by tagging it with a short peptide at the C‐terminal end of CRY2, an outstanding CRY2 clustering system (CRY2clust) was engineered to induce the rapid and efficient homo‐oligomerisation of target proteins in response to light [38]. Light‐induced CRY2 clustering can be used to study protein interaction dynamics and transiently and reversibly control protein function with light. Moreover, CRY2 can also bind CIB1, a basic helix‐loop‐helix (bHLH) protein, in its photoexcited state, dissociating from it in the dark. The dimerisation of CRY2 and CIB1 or CIBN (truncated CIB1 with aa 1–170) is reversible within minutes and triggers protein translocation to the plasma membrane or mitochondria on a sub‐second time scale. This has been used to induce protein translocation, transcription, and Cre‐mediated DNA recombination using light.

In contrast to UV and blue light, which have low tissue penetrance and high toxicity, red/far‐red light is more attractive for optoelectronic applications. Chrimson is a red light (625 nm)‐activated channelrhodopsin with spectra that are red‐shifted by 45 nm compared to those of other channelrhodopsins [23]. It has been utilised together with another channelrhodopsin, chromos, for the two‐colour‐independent optical excitation of distinct neural populations in the mouse brain. Phytochromes are a family of sensory photoreceptors (designated phyA through phyE) found in plants in two forms that are reversibly interconvertible in response to light [39]. The biologically inactive Pr form is converted to the biologically active Pfr form after absorbing red light, which is converted back to the Pfr form after absorbing far‐red light (FRL). Phytochromes A and B (PhyA and PhyB) from *Arabidopsis thaliana* are utilised to construct optogenetic switches based on their interaction with partner proteins. Upon illumination with red light, the Pfr form of PhyB binds to the phytochrome interacting factor (PIF3/PIF6), and the complex dissociates under the stimulus of FRL [40]. The interaction between PhyA and the shuttle protein far‐red elongated hypocotyl 1 (FHY1) is based on the same principle [41]. Notably, the interaction between and dissociation of PhyB‐PIF3/PIF6 and PhyA‐FHY1 occur within milliseconds [42]. However, the chromophore phycocyanobilin (PCB) is required for PhyA/PhyB to respond to light, which is absent in mammalian cells. This requires addition of exogenous PCB or expression of genes for PCB synthesis.

A near‐infrared (NIR) light‐activated BphP1–PpsR2 system was constructed using BphP1 (bacteriophytochrome P1), a photosensitive protein from *Rhodopseudomonas palustris* [43]. Similar to that with phytochromes, there are also two conformational states (Pr and Pfr) of bacterial phytochromes that absorb far‐red and NIR light, respectively. BphP1 and PpsR2 dissociate in the dark and associate with each other upon illumination with NIR light. The interaction between BphP1 and PpsR2 is inhibited either with FRL or through thermal relaxation in the dark [44]. BphP1‐based systems are characterised by rapid (sub‐second) response and reversibility; however, their applications are limited by the large size, multidomain structure, and oligomeric behaviour of PpsR2. Subsequently, a single‐domain BphP1‐binding partner, Q‐PAS1, was engineered by truncating PpsR2 [45]. This has a molecular mass of 17 kDa and is three‐fold smaller than that of wild‐type PpsR2, and lacks oligomerisation capacity. An NIR‐light‐controllable BphP1‐Q‐PAS1 system was also constructed with a 40‐fold increase in transcriptional activation in cells, and it was utilised to modify the chromatin epigenetic state. It is ff note that biliverdin, a chromophore, is required for the light‐sensing capacity of BphP1, which is naturally present in mammalian cells.

BphG1 is a bacteriophytochrome from *Rhodobacter sphaeroides* that contains an N‐terminal photoreception domain, a diguanylate cyclase (DGC) domain, and a phosphodiesterase (PDE) domain, of which DGC and PDE are involved in the synthesis and degradation of the bacterial second messenger cyclic diguanylate monophosphate (c‐di‐GMP), respectively [46]. In contrast to the holoprotein with only c‐di‐GMP PDE activity, a truncated derivative of BphG1 (BphG) has low DGC activity. It is converted from the Pr form to the Pfr form upon irradiation with red light [47]. To increase DGC activity, the low‐activity GGDEF domain of BphG was replaced with a more active one from *Synechocystis* sp. Slr1143 and the conserved RXXD sequence motif located in the GGDEF domain were further mutated (R587A), resulting in an engineered bacteriophytochrome BphS with higher c‐di‐GMP synthase activity and the same photochemical properties as BphG [47]. Stimulator of interferon genes (STING) in mammalian cells functions as a cyclic di‐nucleotide sensor that can detect the presence of c‐di‐GMP, and BphG and BphS have been introduced to construct optogenetic systems based on c‐di‐GMP‐dependent STING activation [8, 48]. It is of significance that BphG/BphS‐based optogenetic systems can be used for the orthogonal regulation of biological activities in mammalian cells in which c‐di‐GMP is not synthesised. There are also other optogenetic tools such as B12‐based systems and multichromatic systems. The partially photosensitive elements and their properties are summarised in Table 1.

**TABLE 1:** Summary of photosensitive elements

Photosensitive elements	Chromophore	Wavelength of excitation/reversion (nm)	Excitation time	Reversion time	Size (aa)	Source	Mechanism of light response	References
UVR8‐COP1	Internal Trp285 and Trp233	382/dark	milliseconds	hours	440–675	*Arabidopsis thaliana*	Heterodimerisation	[20]
FKF1‐GI	FMN	450/dark	milliseconds	hours	619–1173	*A. thaliana*	Heterodimerisation	[35]
Magnets	FAD	450/dark	seconds	seconds to hours	150–152	*Neurospora crassa*	Heterodimerisation	[33]
CRY2‐CIB1	FAD	450/dark	seconds	minutes	535‐170	*A. thaliana*	Heterodimerisation	[36]
PhyB‐PIF	PCB	660/740	milliseconds	milliseconds	908‐100	*A. thaliana*	Heterodimerisation	[40]
PhyA‐FHY1	PCB	660/740	milliseconds	milliseconds	617‐201	*A. thaliana*	Heterodimerisation	[41]
BphP1‐PpsR2	Biliverdin	760/640	seconds	seconds	732‐465	*Rhodopseudomonas palustris*	Heterodimerisation	[44]
EL222	FMN	450/dark	seconds	seconds	225	*Erythrobacter litoralis*	Homodimerisation	[34]
VVD	FMN/FAD	450/dark	seconds	hours	186	*Neurospora crassa*	Homodimerisation	[32]
DrBphP	Biliverdin	660/780	—	—	755	*Deinococcus radiodurans*	Homodimerisation	[43]
CRY2	FAD	450/dark	seconds	milliseconds	535	*A. thaliana*	Homo‐oligomerisation	[7]
PhoCl	—	380/—	minutes	Irreversible	242	*Clavularia* sp.	Photocleavage	[21]
ChR2	Retinal	450/dark	milliseconds	milliseconds	737	*Chlamydomonas reinhardtii*	Ion channel	[1]
Melanopsin	Retinal	450/dark	milliseconds	seconds	534	Mammalian retina	Ion channel	[13]
Chronos	Retinal	470/dark	—	—	326	*Stigeoclonium helveticum*	Ion channel	[23]
Chrimson	Retinal	590/dark	—	—	351	*Chlamydomonas noctigama*	Ion channel	[23]
AsLOV2	FMN	450/dark	seconds	Tens of seconds	143	*Avena sativa*	Intramolecular conformational change	[29]
BcLOV4	FMN	450/dark	seconds	minutes	593	*Botrytis cinerea*	Intramolecular conformational change	[15]
cpLOV2	FMN	450/dark	—	—	143	*A. sativa*	Intramolecular conformational change	[30]
bPAC	FAD or FMN	450/dark	seconds	Tens of seconds	483	*Beggiatoa* sp.	cAMP production	[82, 83]
BphG	Biliverdin	660/760	—	—	508	*Rhodobacter sphaeroides*	c‐di‐GMP production	[47]
BphS	Biliverdin	660/760	—	—	681	*Rhodobacter sphaeroides, Synechocystis* sp. Slr1143	c‐di‐GMP production	[47]

### 1.2 Optocontrolled genome engineering

With the development and improvement of optogenetic tools, they have been applied in various fields, including biomedical engineering, with high accuracy and in accordance with safety requirements [49]. The accurate manipulation of genes has important implications for parsing complex living systems and developing precision medicine [50]. Synthetic biology provides a powerful platform to design and harness optogenetic circuits, which have broad application prospects in gene editing and epigenome regulation, including gene recombination, gene editing, and transcriptional regulation.

#### 1.2.1 Gene recombination

**FIGURE 1:**
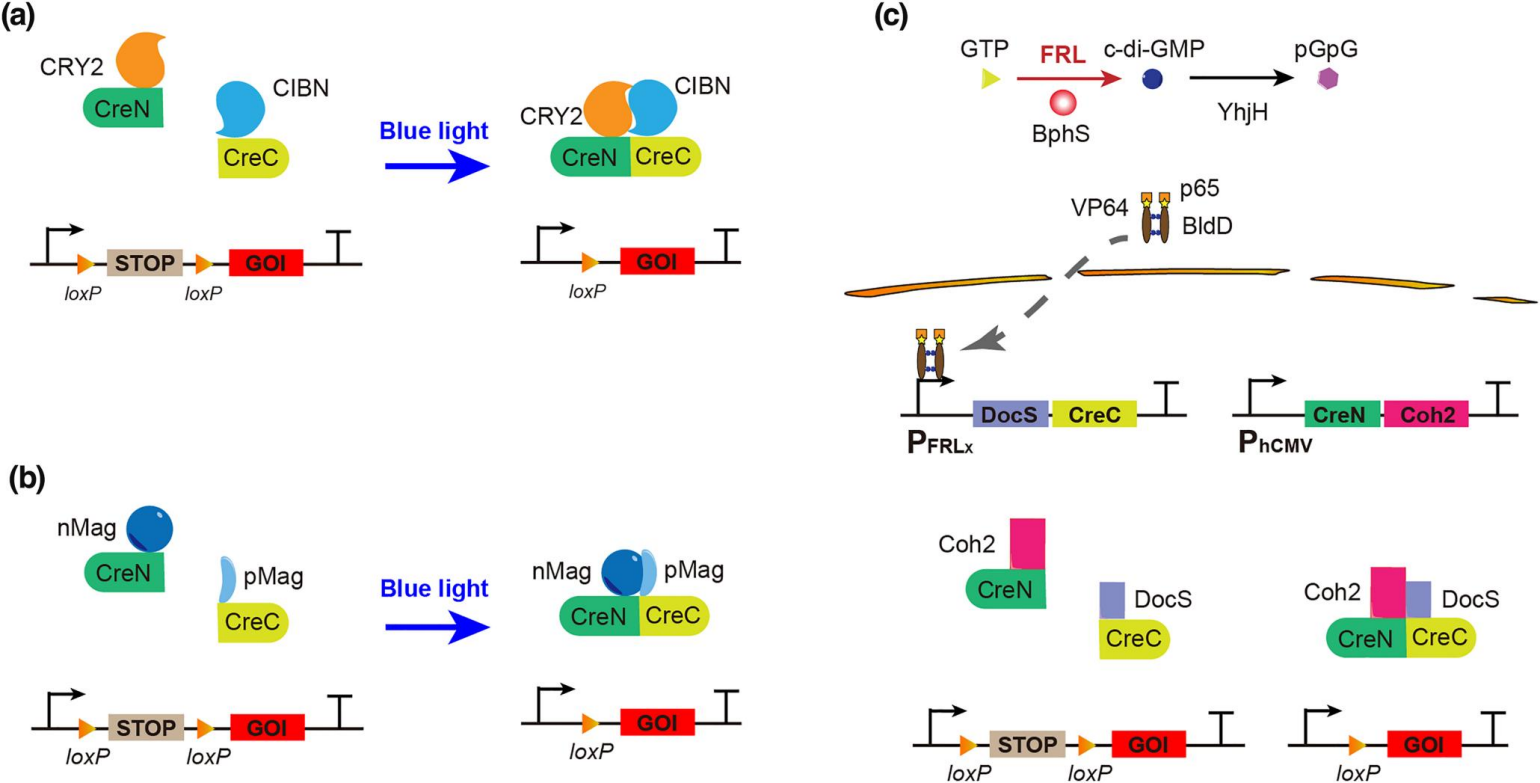
Optocontrolled Cre recombinase system for gene recombination. (a) Blue light inducible CRY2‐Cre system. Under blue light illumination, CRY2 changes its conformation and binds to CIBN, thereby forcing CreC and CreN to form a complete and active Cre recombinase. (b) Blue light inducible PA‐Cre system. pMag and nMag combine under blue light illumination to trigger CreC and CreN to form a complete and active Cre recombinase. (c) BphS‐based FRL inducible Cre‐*loxP* system. Under FRL illumination, the photoreceptor BphS converts intracellular GTP into c‐di‐GMP, leading to the dimerisation of the hybrid transcriptional activator p65‐VP64‐BldD, which is transported into the nucleus and activates expression of the DocS‐CreC fusion protein. The expression of the CreN‐Coh2 fusion protein is constitutively activated by the CMV promoter. CreC and CreN combine to form a complete and active Cre recombinase due to the interaction of DocS and Coh2, which interact spontaneously

Site‐specific DNA recombinase is a powerful gene‐manipulation tool in genome engineering, in which Cre recombinase is most widely used to catalyse DNA recombination between two pairs of 34 bp recognition sites (*loxP* sites) [51]. Light‐inducible Cre‐*loxP* systems have been developed to improve the precision of gene recombination, including Cre‐*loxP* systems induced by UV [52, 53], blue light [54, 55], and FRL [56]. All blue light‐inducible Cre‐*loxP* systems rely on the split‐Cre recombinase concept. Initially, the PA (photoactivatable)‐Cre system was constructed by fusing CRY2 with amino acids 19–104 of Cre (CRY2‐CreN) and CIBN with amino acids 106–343 of Cre (CIBN‐CreC) [36]. The reporter contains a transcriptional stop sequence flanked by *loxP* sites that precede the target gene. Exposure to pulsed blue light (461 nm) leads to the dimerisation of CRY2 and CIBN, reconstitution of Cre recombinase activity, and significant activation of gene expression (Figure 1a). One hour of exposure was found to result in approximately a 50‐fold increase in gene expression in cells compared to that in samples maintained in the dark. This PA‐Cre system has also been used to permanently modify gene expression in the mouse brain [57]. Subsequently, a second‐generation PA‐Cre system was engineered by substituting CRY2(L348F) for CRY2 in the CRY2‐CreN fusion, which resulted in a 50% reduction in the levels of recombination in the dark and a 35% increase in Cre recombinase activity in cells [54].

Simultaneously, magnet‐based PA‐Cre was developed by fusing Cre [19, 20, 21, 22, 23, 24, 25, 26, 27, 28, 29, 30, 31, 32, 33, 34, 35, 36, 37, 38, 39, 40, 41, 42, 43, 44, 45, 46, 47, 48, 49, 50, 51, 52, 53, 54, 55, 56, 57, 58, 59] to nMag (CreN59–nMag) and Cre (60–343) to pMag (pMag–CreC60) (Figure 1b), which enabled efficient DNA recombination in mammalian cells (up to 320‐fold induction) [55]. The high recombination efficiency using PA‐Cre substantially outperforms that of the CRY2–CIB1 split Cre system, allowing for DNA recombination in mice with illumination for only a short period (∼30 s). Later, by optimising the promoter and 2A self‐cleaving peptide and modifying the CreN‐nMag codons, the magnet‐based PA‐Cre 3.0 was designed with reduced leaky activity in the dark and improved blue light induction [58]. The PA‐Cre 3.0 adeno‐associated virus (AAV) was used to target active neurons in mice.

Recently, an FRL‐induced Cre‐*loxP* (FISC) system was constructed based on split‐Cre recombinase and an FRL‐triggered optogenetic system [56]. An FRL‐triggered optogenetic system was constructed based on BphS, an FRL‐activated c‐di‐GMP synthase derived from *R. sphaeroides*, and BldD, a *Streptomyces coelicolor*‐derived transcription factor [8]. BphS converts intracellular guanylate triphosphate (GTP) to c‐di‐GMP under FRL irradiation. The increase in c‐di‐GMP triggers dimerisation of the artificial hybrid transactivator FRTA (p65‐VP64‐BldD), and the BldD dimer specifically binds to DNA operator sequences containing the *whiG* motif. Dimeric p65‐VP64‐BldD is translocated into the nucleus to bind to the FRTA‐specific chimaeric promoter P_FRL_ to drive transgene expression. To decrease basal activation, the c‐di‐GMP‐specific phosphodiesterase YhjH was expressed to reduce basal intracellular c‐di‐GMP production. The Cre recombinase was split into two fragments at the CreN59/CreC60 residue. CreN59 was fused to the Coh2 domain and constitutively driven by P_hCMV_, whereas CreC was fused to the DocS domain and induced by P_FRL_. Upon FRL illumination (730 nm), DocS‐CreC60 was expressed, and the activity of Cre recombinase was reconstituted owing to the strong affinity of Coh2 for DocS (Figure 1c). Due to the negligible phototoxicity, high spatiotemporal specificity, and deep‐tissue penetrative capacity of FRL, this system results in high DNA recombination efficiency in vitro and in vivo. Notably, after illumination for 12 h, there was an approximate 54‐fold increase in DNA recombination in mice hydrodynamically injected with the FISC system, whereas the maximum increase in mice bearing the CRY2‐Cre or PA‐Cre system was found to be only three‐fold [56].

#### 1.2.2 Gene editing

The revolutionary CRISPR‐Cas9 gene‐editing system has garnered much attention in recent years owing to its high efficiency, ease of operation, and low cost [59]. Therefore, it has been widely applied in functional genomic research and genetic disease treatment. Nevertheless, the well‐known disadvantages of the CRISPR‐Cas9 system, such as off‐target effects owing to the lack of Cas9 activity control, have led to serious safety hazards in its clinical application [60]. Controllable CRISPR‐Cas9 systems have been developed to overcome such challenges, and the activity or lifetime of Cas9 has been regulated to reduce the interaction time between the Cas9 nuclease and the genome.

**FIGURE 2:**
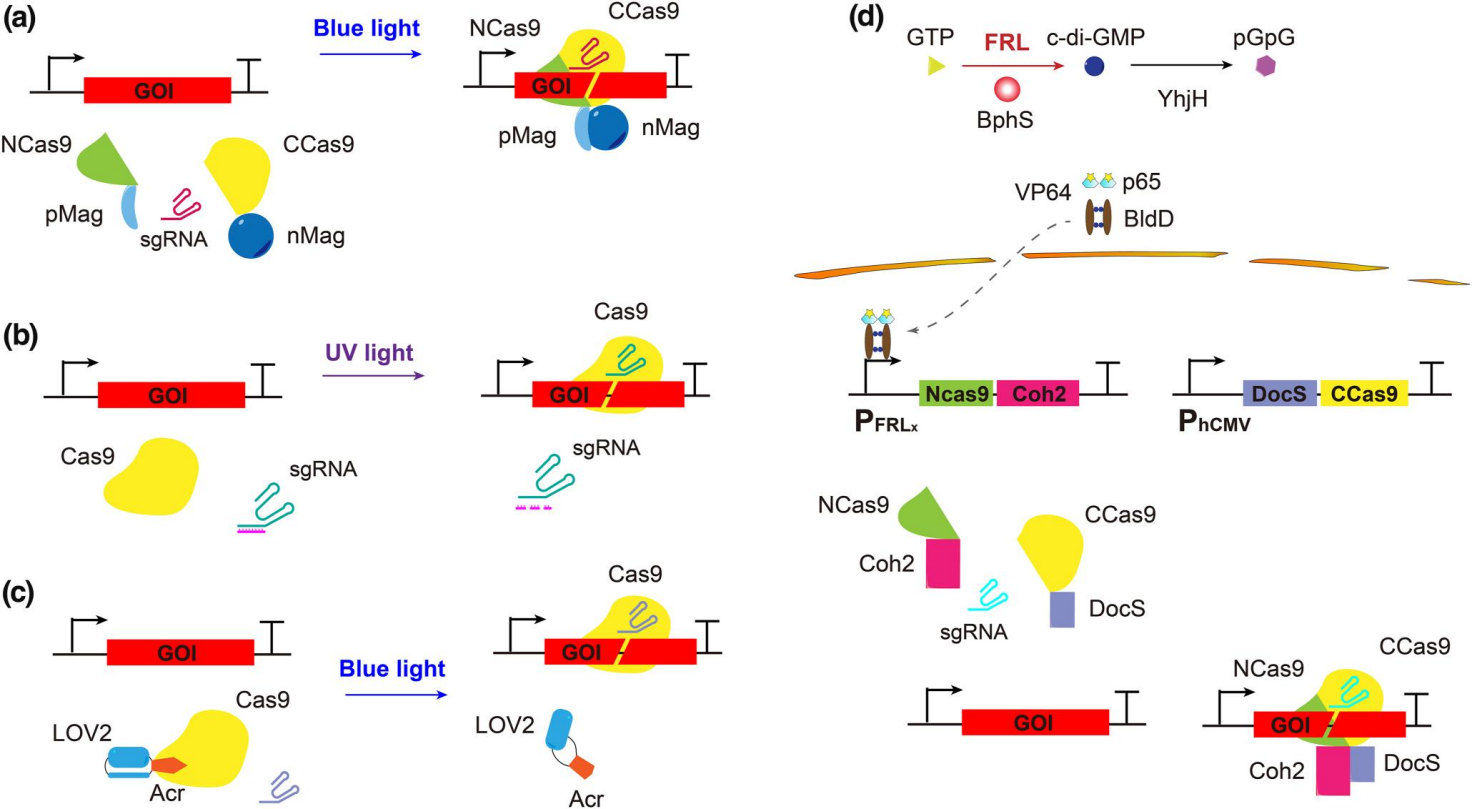
Optocontrolled CRISPR‐Cas9 system for gene editing. (a) Blue light inducible Cas9 system based on pMag/nMag. pMag and nMag combine under blue light illumination, promoting NCas9 and CCas9 to form a complete and active Cas9 nuclease for gene editing. (b) UV light‐induced CRISPR‐Cas9 system based on protected‐sgRNAs. The seed sequences of sgRNA are bound by an oligonucleotide chain, which is cleaved by UV light, and thus, sgRNA is exposed to guide Cas9 to cleave DNA. (c) Blue light mediated anti‐CRISPR system based on LOV2 and AcrIIA4. The AcrIIA4‐LOV2 complex binds to Cas9 to inhibit its activity in the dark, which is separated under blue light illumination and the nuclease activity of Cas9 is restored. (d) BphS‐based FRL inducible CRISPR‐Cas9 system. Under FRL illumination, the photoreceptor BphS converts intracellular GTP into c‐di‐GMP, resulting in dimerisation of the hybrid transcriptional activator p65‐VP64‐BldD, which is transported into the nucleus and activates expression of the NCas9‐Coh2 fusion protein. The expression of the DocS‐NCas9 fusion protein is constitutively activated by the CMV promoter. NCas9 and CCas9 combine to form a complete and active Cas9 nuclease due to the interaction of DocS and Coh2, which interact spontaneously

Compared to chemical compound‐induced CRISPR‐Cas9 systems with potential cytotoxicity and low spatial resolution [61, 62], the optical control of Cas9 activity has significant advantages, including reversibility and spatiotemporal regulation [63]. In 2015, optogenetic methods were first used to construct a photoactivatable Cas9 system, in which amino acids 2–713 of Cas9 were fused to pMag and nMagHigh1 (nMag with M135I and M165I mutations) was fused to amino acids 714–1368 of Cas9 (Figure 2a). Activated Cas9 nucleases were reconstituted in HEK293T cells through the interaction between pMag and nMag induced by blue light (470 ± 20 nm) illumination, and the targeted genome sequence was modified through both non‐homologous end‐joining and homology‐directed repair pathways [64].

A UV‐inducible CRISPR‐Cas9 system named “CRISPR‐plus” was designed to achieve gene editing in cells by photocaging the guide RNA [65]. Specifically, complementary ssDNA oligonucleotides were designed as protectors to hybridise to the target region of sgRNA, preventing it from binding to the target DNA. Upon illumination (365 nm) for only 2–5 s, the protector was photolysed and released from the sgRNA, resulting in Cas9‐mediated cleavage of the target DNA (Figure 2b).

Later, a blue‐light‐mediated anti‐CRISPR system was developed based on LOV2 and the Cas9 inhibitor AcrIIA4 [66]. The Acr‐LOV hybrid bound to Cas9‐sgRNA complexes blocks Cas9 DNA‐binding and nuclease activity in the dark. Under blue light (488 nm) illumination, the unfolding of the alpha‐helix adjacent to LOV2 leads to the dissociation of AcrIIA4 from Cas9, the activity of which is recovered (Figure 2c). Anti‐CRISPR has been utilised for the optogenetic control of gene expression and telomere labelling in human cells.

A CRISPR‐Cas9 gene editing system (FAST) regulated by FRL with deep tissue penetration was constructed based on the P_FRL_ system [8]. It comprises two separate (N‐ and C‐terminal) Cas9 fragments and Coh2‐DocS protein pairs. Inactive Cas9 (N) and Cas9 (C) were fused with Coh2 and DocS, respectively. The DocS‐Cas9 (C)‐NES fusion protein is constitutively expressed, whereas expression of the Cas9 (N)‐Coh2 fusion protein is induced by FRL [67]. Complete Cas9 was also reconstituted to achieve gene editing activity under FRL illumination (730 nm) via LED (Figure 2d). The FAST system has been shown to achieve programmable deep‐tissue gene editing in mice at a frequency of ∼21.5%.

#### 1.2.3 Gene transcriptional regulation

**FIGURE 3:**
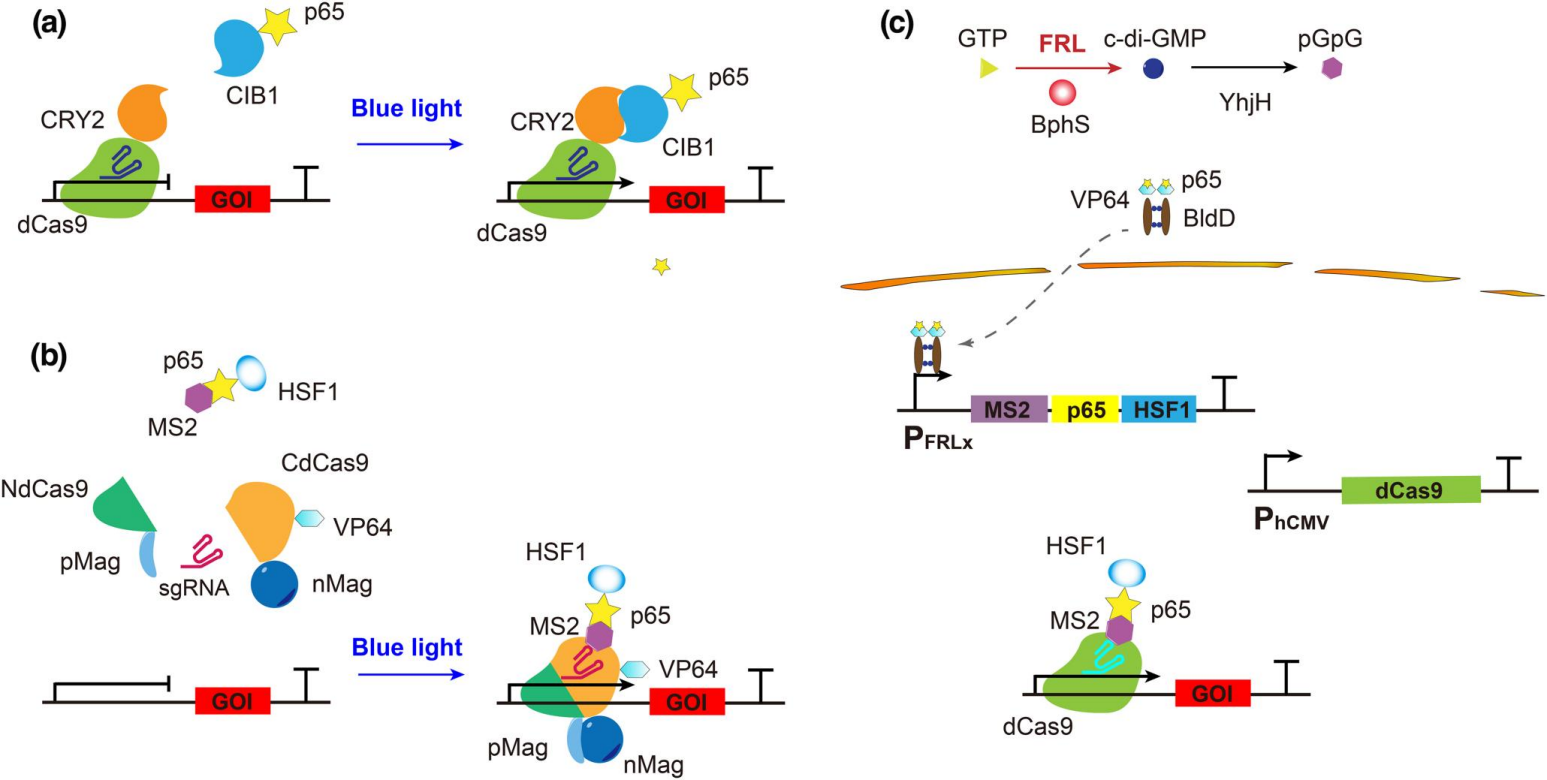
Optocontrolled CRISPR‐dCas9 system for gene transcriptional regulation. (a) A blue light inducible CRISPR‐dCas9 system based on CRY2/CIBN. dCas9 and the transcriptional activator P65 are fused to CRY2 and CIB1, respectively, and dCas9‐CIB1 binds to the target DNA with the guidance of sgRNA. Under blue light illumination, CRY2 undergoes a conformational change and binds to CIB1, thereby bringing P65 to the target DNA and activating gene expression. (b) Blue light‐inducible CRISPR‐dCas9 system based on pMag/nMag. pMag and nMag combine under blue light illumination, promoting NdCas9 and CdCas9 to form a complete and active dCas9. Meanwhile, the MS2‐box sequence recognised by the MS2 protein is inserted into the loop structure of sgRNA, which enables the MS2 protein to fuse with the hybrid transcriptional activator P65‐HSF1 to activate transcription. (c) BphS‐based FRL inducible CRISPR‐dCas9 system. Under FRL illumination, the photoreceptor BphS converts intracellular GTP into c‐di‐GMP, resulting in dimerisation of the hybrid transcriptional activator p65‐VP64‐BldD, which is transported into the nucleus and activates the expression of MS2‐p65‐HSF1, further activating gene transcription through the binding of sgRNA and dCas9

dCas9 (dead Cas9) is a powerful tool for manipulating endogenous gene expression owing to its functions in activating or inhibiting the transcription of specific genes when combined with a transcriptional activator or repressor [68]. In 2015, Nihongaki et al. constructed a CRISPR‐dCas9 transcriptional control system (CPTS) triggered by blue light, based on CRY2‐CIB1 [69]. It consists of a dCas9‐CIB1 fusion protein formed by the photolyase homology region of CRY2 (CRY2PHR), a transcriptional activator (P65), and sgRNAs. dCas9‐CIB1 binds to the targeted DNA sequence under sgRNA guidance. When exposed to blue light (470 ± 20 nm), CRY2PHR and CIB1 heterodimerise to recruit transcriptional activators to activate target gene expression (Figure 3a). CPTS allows for rapid and reversible activation of endogenous target genes (∼50‐fold for *ASCL1*) in mammalian cells. Simultaneously, a similar light‐activated CRISPR‐dCas9 effector system was engineered by fusing CRY2 to a transactivation domain (VP64) and CIBN to both dCas9 termini [70]. CIBN‐dCas9‐CIBN localises to the DNA sequence targeted by the gRNA in the dark, whereas under blue light illumination (450 nm), the interaction between CRY2 and CIBN results in the translocation of CRY2‐VP64 to the targeted DNA sequence to initiate transcription of the downstream gene. It has been proven effective for the transcriptional activation of multiple endogenous genes in mammalian cells, which results in mRNA levels that are hundreds of times greater after illumination.

Later, an improved CRISPR‐dCas9 transcriptional activation system (Split‐CPTS 2.0) was developed for more potent activation, which consists of NES‐dCas9_N_‐pMag‐NES, nMagHigh1‐dCas9_C_‐NLS‐VP64, and NLS‐MS2_ΔFG_‐NLS‐p65‐HSF1 [71]. Here, activated dCas9 is reassociated as a heterodimerisation partner of pMag and nMag under blue light (470 nm) illumination (Figure 3b). This system has also been applied to induce neuronal differentiation in induced pluripotent stem cells (iPSCs) and functions by upregulating neurogenic differentiation factor 1 (*NEUROD1*). However, the slow dissociation kinetics of split dCas9 fragments and/or the Magnet system lead to a slow switching‐off rate of Split‐CPTS2.0. For faster reversible dCas9‐targeted gene regulation, CPTS 2.0 was developed based on full‐length dCas9 and CRY2‐CIB1, which consists of dCas9, sgRNA with MS2 aptamers fused to CIB1, and CRY2 fused to p65 and HSF1 activator domains [71]. It demonstrated robust, reversible and repeated transcriptional light regulation of endogenous genes in mammalian cells.

Similarly, to overcome the shortcomings of blue light, an FRL‐mediated CRISPR‐dCas9 device (FACE) was engineered to induce the transcription of exogenous or endogenous genes [72]. It also relies on the BphS‐based FRL‐regulated transgenic expression system. The transactivators p65 and HSF1 were fused to MS2 and induced via FRL (730 nm) (Figure 3c). The sgRNA‐mediated recruitment of the MS2‐p65‐HSF1 complex results in the transcriptional activation of dCas9‐targeted endogenous genes. The FACE system can be utilised to photoactivate user‐defined endogenous genes not only in mammalian cells but also in mice, and the endogenous gene activation efficiency of the FRL‐controlled FACE system is significantly higher than that of the blue‐light‐controlled CPTS2.0 system. Specifically, expression of the endogenous *Lama1* or *Fst* gene in muscle tissues can be significantly upregulated by the FACE system but cannot be upregulated by CPTS2.0, compared to that in mice left in the dark. Furthermore, the FACE system was applied to induce the neuronal differentiation of iPSCs with sgRNAs targeting neurogenin 2 (*NEUROG2*). These optocontrolled systems are potentially applicable for optoregulation of gene expression in disease treatment.

### 1.3 Optogenetic circuits and optomedicine

With the development of synthetic biology technology, gene‐ and cell‐based therapies have become the focus of a new generation of biomedicine [73]. By combining synthetic biology with optogenetics, precision therapies have been developed to reduce the off‐target pharmacological intervention and improve the spatiotemporal specificity of gene‐ and cell‐based therapies [12]. By linking synthetic biology, optogenetics and electronic information technology, it is possible to combine disease monitoring and treatment [8]. Currently, multiple optogenetic circuits have been constructed to treat serious diseases, such as diabetes, cancers, cardiovascular diseases, and neurological diseases [9, 74, 75, 76]. The potential of optogenetic circuits for the treatment of diseases, such as diabetes and cancer, is elaborated in further sections.

#### 1.3.1 Phototherapy for diabetes

Diabetes is a serious metabolic disease that severely affects human health [77]. However, current treatments, while efficacious, have side effects and require patient compliance [78]. In particular, the excessive delivery of insulin can lead to hypoglycemia and even death. Light‐controlled cell therapy represents a new strategy for diabetes treatment. Here, we highlight the latest progress in the use of optogenetic circuits to lower blood glucose and discuss the potential applications of optogenetics for future phototherapy for diabetes.

**FIGURE 4:**
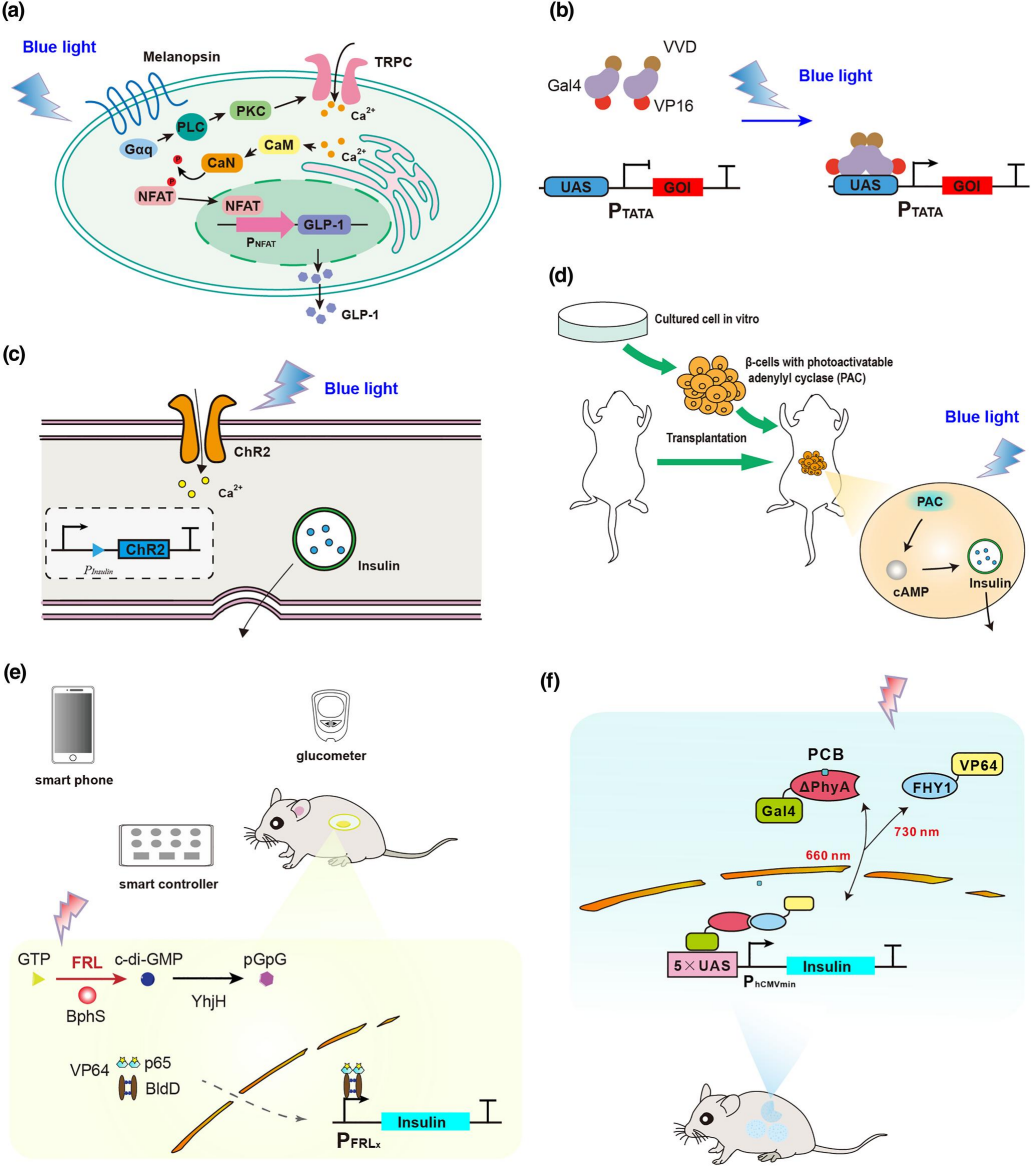
Optogenetic circuits developed for diabetes therapy. (a) Melanopsin‐based blue light‐activated GLP‐1 expression system. The conformation of melanopsin changes upon blue light illumination, thereby activating G protein (GαQ), phospholipase C (PLC), and phosphate kinase C (PKC), leading to the opening of transient receptor potential ion channels (TRPCs) and intracellular organelle (endoplasmic reticulum) calcium channels and causing a rapid influx of calcium ions. NFAT is then phosphorylated and transported into the nucleus, and the promoter responding to NFAT (P_NFAT_) is activated to express GLP‐1. (b) VVD‐based blue light‐controlled transgene expression system. The photosensitive VVD protein dimerises after it is stimulated by blue light, and the associated Gal4 recognition domain (1–65 aa) dimerises, is translocated into the nucleus, and binds to the upstream activating sequence (UAS). The transcriptional activator VP16, fused with it, recruits transcription factors to activate the expression of insulin. (c) ChR2‐based blue light‐controlled insulin release system. ChR2 is introduced into islet cells and is activated by blue light to mediate the influx of Ca^2+^, thereby causing the release of insulin stored in vesicles. (d) Light‐sensitive adenylate cyclase‐based blue light‐controlled insulin release system. Adenylate cyclase is activated by blue light, leading to an increase in intracellular cAMP and the release of insulin. (e) Semi‐automatic intelligent diagnosis and treatment system based on FRL‐controlled customised cells. Blood glucose is measured by a glucose metre, which can be automatically transmitted to the smart controller and smart phone through Bluetooth technology. Different intensities of FRL will be delivered by the smart controller according to the preset programme. Under FRL illumination, the photoreceptor BphS in customised cells is transplanted subcutaneously, converts intracellular GTP into c‐di‐GMP, and results in the dimerisation of the hybrid transcriptional activator p65‐VP64‐BldD, which is transported into the nucleus and binds to the chimaeric promoter to express insulin. (f) Red/far‐red light‐mediated and miniaturised Δphytochrome A (ΔPhyA)‐based photoswitch (REDMAP) system. Microencapsulated HEK293 cells containing the REDMAP system were subcutaneously implanted under the dorsum of diabetic mice or rats. When illuminated by red light (660 nm), the transactivator (FHY1–VP64) can specifically bind to the light sensor (ΔPhyA–Gal4) in the presence of PCB, translocate into the nucleus, and bind to the synthetic promoter to express insulin and promote its release

Optogenetic technology was developed in 2011 to control blood glucose homoeostasis in a diabetic mouse model [5]. Researchers have designed a synthetic signalling cascade by functionally linking the signal transduction of melanopsin to the control circuit of the nuclear factor of activated T cells (NFAT). The conformational changes in melanopsin induced by blue light (∼480 nm) lead to Ca^2+^ influx and activate calcineurin, causing NFAT dephosphorylation and nuclear translocation (Figure 4a). NFAT‐regulated glucagon‐like peptide 1 (GLP‐1) expression provides glycaemic control in type 2 diabetic mice [5]. A VVD‐based blue‐light‐switchable transgene system was developed and applied to enhance blood glucose homoeostasis in type 1 diabetic mice [79]. The hybrid transactivator consisting of the DNA‐binding domain of Gal4, VVD, and transcriptional activator VP16 homodimerise upon blue‐light exposure and bind the artificial promoter to initiate the transcription of insulin (Figure 4b). A large decline in blood glucose levels (from more than 25 mM to approximately 10 mM) was found to be induced by blue light illumination (460 nm) in diabetic mice.

Because insulin is secreted from pancreatic *β*‐cells, optogenetically controlled insulin secretion systems have been designed in *β*‐cells based on ChR2. Reinbothe et al. used the insulin promoter to express ChR2 in transgenic mice, which was activated under blue light (470 nm), resulting in the influx of Ca^2+^ and the release of insulin (Figure 4c) [80]. Light illumination of islets from mice that were fed a high‐fat diet was found to significantly potentiate insulin release at both low and high levels of glucose. Kushibiki et al reported that ChR2 was stably expressed in the MIN6 cell membrane, and the increased intracellular Ca^2+^ triggered by blue light stimulation (460 nm) led to enhanced insulin secretion [81]. A 30% decrease in blood glucose levels was observed in diabetic mice 30 min after blue light irradiation for 10 s. However, increasing Ca^2+^ in *β*‐cells can lead to the release of insulin independent of extracellular glucose, resulting in over‐ or under‐regulation of blood glucose levels. Cyclic adenosine monophosphate (cAMP) is one of the intermediates that regulate the release of insulin in pancreatic *β*‐cells, ensuring homoeostasis of blood glucose. Photoactivatable adenylyl cyclase was also introduced into pancreatic *β*‐cells, which induced increases in intracellular cAMP levels under blue light (465 nm) irradiation and enhanced glucose‐stimulated insulin secretion (Figure 4d) [82, 83].

In 2017, a smartphone‐controlled system was constructed to monitor and regulate blood glucose levels in diabetic mice [8]. It was developed by combining optogenetically engineered cells with software and electronic engineering. An FRL‐triggered optogenetic system was based on BphS and p65‐VP64‐BldD (Figure 4e), and FRL‐controlled customised cells engineered with this optogenetic switch were implanted in diabetic mice together with wirelessly powered FRL (∼730 nm) LEDs. The FRL was adjusted using a smartphone according to the blood glucose signal, which was automatically delivered from the customised glucometer using Bluetooth technology, and insulin or GLP‐1 was expressed and released to maintain blood glucose homoeostasis in type 1 or type 2 diabetic mice. An integrated system of monitoring and treatment is poised for clinical translation. FRL‐activated human islet‐like designer (FAID) cells have been developed for long‐term blood glucose control [84]. The FRL‐triggered optogenetic device was introduced into human mesenchymal stem cells, encapsulated in poly(l‐lysine)‐alginate, and implanted subcutaneously under the dorsum of type 1 diabetic mice. Improved glucose tolerance and sustained blood glucose control were achieved through FAL illumination (730 nm) for 2 h/day. Moreover, oxidative stress and multiple diabetes‐related complications in the kidneys were attenuated by FAID cell therapy.

Recently, a new red/far‐red optogenetic switch was developed, based on the plant photoreceptor PhyA that binds the phycocyanobilin (PCB) chromophore [41]. The yeast Gal4 DNA‐binding domain and transactivator VP64 were fused with PhyA and FHY1, respectively. Under red light illumination (660 nm), PhyA interacts with FHY1, resulting in binding of the transcription factor to the synthetic promoter to initiate transcription. Under FRL illumination (730 nm), the PhyA‐FHY1 interaction is disrupted, the transactivator dissociates from the promoter, and expression of the target transgene is terminated. For in vivo applications, a PCB‐producer plasmid for the constitutive expression of four enzymes, which together produce PCB starting from heme, was transfected into designer cells together with the optogenetic system. Glucose homoeostasis was successfully maintained in type 1 diabetic mice and rats implanted with customised cells using this compact and sensitive tool, for which insulin expression could be controlled by illumination for 1 or 5 min twice per day with 660 nm LED light. Nevertheless, the clinical applications of these optogenetic systems are hindered by several bottlenecks including the immunogenicity of genetic circuits, limited tissue penetration by light, and restricted life span of designer cells. Therefore, extensive improvements and breakthroughs are essential for phototherapy to replace current treatments for diabetes.

#### 1.3.2 Photoimmunotherapy for cancer

**FIGURE 5:**
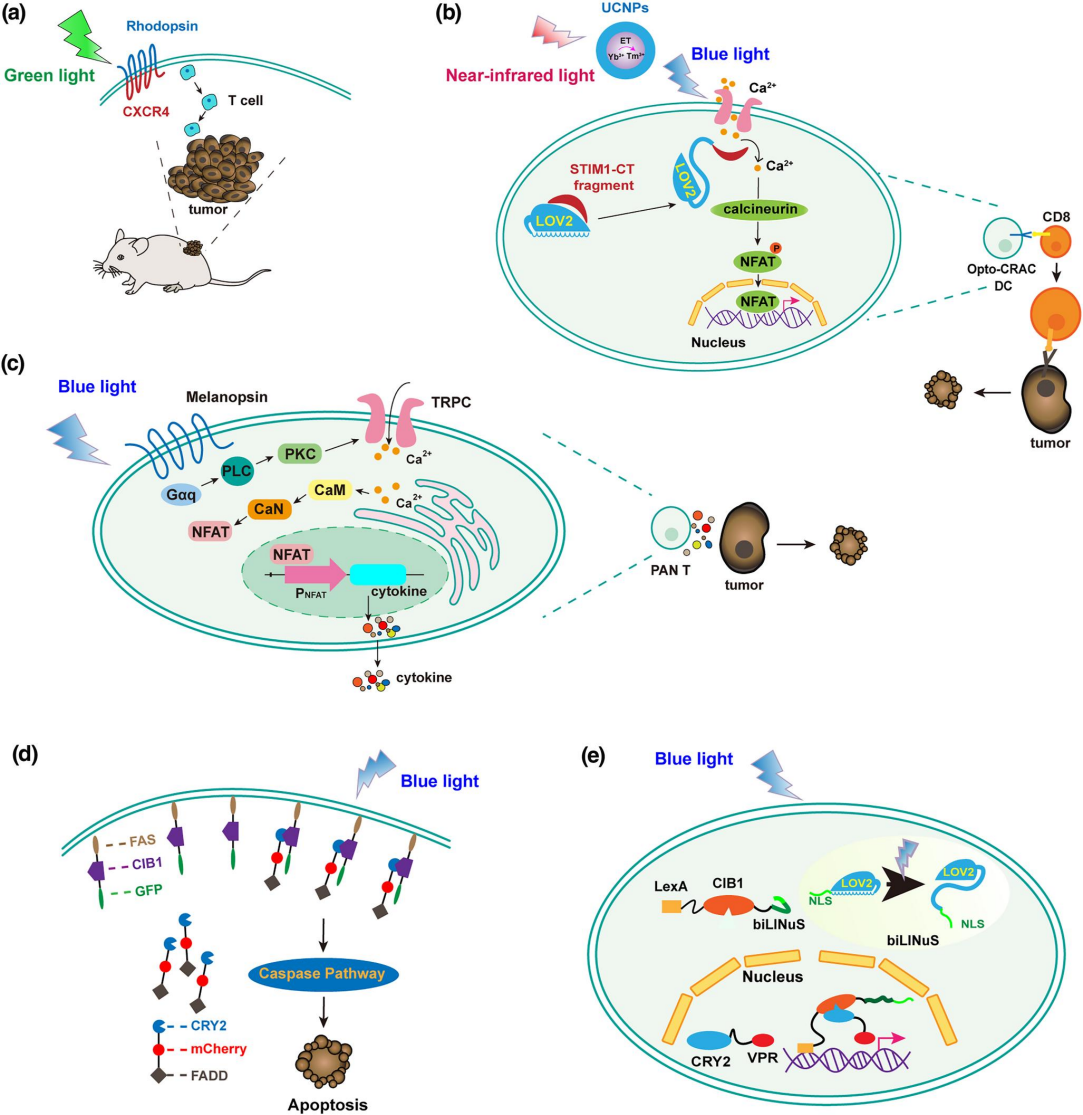
Optogenetic circuits developed for tumour immunotherapy. (a) Optogenetic control of T cell migration based on chemokine receptors. The photoactivated chemokine receptor consisting of the alpha subunit of rhodopsin and chemokine receptor‐4 (CXCR4) is activated by green light (505 nm) to produce a chemokine signal and induce the polarisation of T cells, which migrate to the tumour to suppress its growth. (b) Immune cell activation system based on Opto‐CRAC. The Jα helix of the carboxyl terminal of the LoV2 domain is unfolded upon blue light stimulation (converted from NIR light via UCNPs) and exposes the C‐terminus of STIM1 protein, which stimulates the ORAI1 Ca^2+^ channel and activates T cells and the immune response. (c) Blue light‐triggered immune signalling circuit based on melanopsin. The conformation of melanopsin changes upon blue light illumination, leading to a rapid influx of calcium ions and activating NFAT to express anti‐tumour cytokines. (d) NIR light‐induced cell apoptosis based on CRY2/CIBN. NIR light is converted to blue light via UCNPs to induce the binding of CRY2 and CIBN, and FADD is thereby translocated to Fas on the plasma membrane to induce cell apoptosis. (e) Light‐inducible nuclear translocation and dimerisation (LINTAD) system for CAR‐T activation. CIB1 is fused with LexA and the bipartite light‐responsive nuclear localisation signal (biLINuS), and CRY2 is fused with VPR. The Jα helix of the LoV2 domain in biLINuS is unfolded upon blue light treatment to expose the NLS, leading to the translocation of LexA‐CIB1 into the nucleus. LexA binds to reporter, and CRY2 binds to CIB1 under blue light illumination, thereby recruiting VPR to trigger the expression of CAR

Immunotherapy has become the treatment of choice for multiple cancers. Tumours are inhibited and ablated through immune activation or relieving immunosuppression. Optogenetic systems have been applied to control innate and adaptive immune cells. Here, we present the latest progress in development of anti‐tumour optogenetic systems and propose their potential applications in cancer photoimmunotherapy. In 2014, a photoactivatable chemokine receptor was designed that couples rhodopsin with the chemokine receptor CXCR4 (Figure 5a), resulting in the light‐controlled transmission of intracellular chemokine signals in T cells [85]. This promoted T cell polarisation and directional migration to tumours upon exposure to 505 nm light and significantly reduced tumour growth. He et al. developed an optogenetic platform to improve antigen‐specific immune responses to specifically destroy tumour cells. The system comprises ORAI1 (a four‐pass transmembrane protein that constitutes the CRAC channel pore‐forming subunit in the plasma membrane)‐activating STIM1‐CT (cytosolic domain of stromal interaction molecule 1) and the LOV2 domain [86]. Under blue light (470 nm) illumination, the C‐terminal Jα helix of LOV2 unfolds to expose STIM1‐CT fragments, which move towards the plasma membrane to engage and activate ORAI1 Ca^2+^ channels, thereby inducing Ca^2+^ influx and transgenic expression via the Ca^2+^/NFAT pathway (Figure 5b). Moreover, to transmit light deep within the tissue, upconversion nanoparticles (UCNPs) were introduced that convert NIR light to local blue light. Consequently, an immune response can be induced by NIR light (980 nm) stimulation to dramatically inhibit tumour growth. Additionally, an upconversion optogenetic nanosystem was designed by combining rare‐earth UCNPs with CRY2‐CIB1, through which the caspase apoptotic signalling pathway in cancer cells could be activated by NIR light (980 nm) in both mammalian cells and mice [87].

A blue light‐inducible artificial immune signalling circuit and transgene expression system were designed to regulate the expression of anti‐tumour cytokines (IL‐2, IL‐15, and TNF‐α) for hepatocellular carcinoma immunotherapy (Figure 5c) [74]. The conformational changes in melanopsin in response to blue light (460 nm) illumination initiated a rapid influx of Ca^2+^ and activated the expression of cytokines, which significantly enhanced the expansion ability and cytolytic activity of primary T cells and showed high efficiency for tumour elimination [74]. A CRY2‐CIB1 based blue light‐triggered optogenetic system was developed to treat uveal melanoma, which is the most common intraocular primary malignancy in adults [88]. Fas is a classic apoptosis signalling pathway molecule, and FADD is its adapter molecule. Reversible blue light‐induced binding pairs between Fas‐CIB1‐EGFP and CRY2‐mCherry‐FADD were cotransfected into tumour cells, and FADD was found to translocate to Fas on the plasma membrane under blue light (488 nm) irradiation to induce cell apoptosis, thereby suppressing the growth of uveal melanoma in mice (Figure 5d).

In recent years, immunotherapy based on chimaeric antigen receptor (CAR)‐expressing T cells, which can effectively recognise and kill target tumour cells, has been widely used in tumour therapy [89, 90]. However, severe immune‐mediated adverse effects such as cytokine release syndrome, tumour lysis syndrome, and neurotoxicity can be caused by the uncontrollable CAR T cell activation [91]. Therefore, there is an urgent need to develop accurately controllable CAR‐T technology for tumour immunotherapy. A light‐inducible nuclear translocation and dimerisation (LINTAD) system has been constructed to control CAR‐T activation by controlling the expression of CAR genes in T cells [11]. Specifically, a LexA DNA‐binding domain was fused to the N‐terminus of CIB1 and a bipartite light‐responsive nuclear localisation signal (biLINuS) was fused to the C‐terminus, whereas an NLS was fused to the N‐terminus of CRY2PHR and the transcriptional activator VPR was fused to the C‐terminus. Under blue light (460 nm) illumination, the conformation of LOV2 in biLINuS changes to expose the NLS motif, leading to the nuclear translocation of LexA‐CIB1, which binds to the LexA‐binding site on the reporter cassette. Simultaneously, CRY2PHR is activated by blue light and binds CIB1, and VPR is recruited to the promoter to activate CAR gene expression (Figure 5e). LINTAD CAR‐T cells were activated by pulsed light, with strong cytotoxicity against target cancer cells both in vitro and in mice.

In general, optogenetic circuits are effective tools to non‐invasively and accurately control immunity for targeted cancer immunotherapy. In addition to optocontrolled CAR‐T cells, optogenetic circuits can be equipped with other immune cells, such as NK cells and macrophages, with broad application prospects for cancer treatment. Further, optogenetic circuits have also been applied in many other fields, such as the control of organelle transport and distribution [92, 93], neurobiology [94, 95], the treatment of cardiovascular [96, 97, 98] and neurological diseases [75], and intelligent bioelectronic medicine for blood glucose control and cardiac pacing and resynchronisation therapies [48, 99].

Notably, some optogenetic approaches have been used in clinical trials for the treatment of inherited retinal diseases. Opsins were selected as therapeutic agents with AAV vectors as delivery vehicles. RetroSense Therapeutics developed an AAV2 optogenetic therapy with ChR2 to treat retinitis pigmentosa, a neurodegenerative eye disease characterised by the deterioration of rod and cone photoreceptors [100]. However, the sensitivity of ChR2 to light is poor, therefore, bright light is required. GenSight Biologics delivered amber‐light‐sensitive opsins (ChrimsonR) via AAV, and the visual function of a blind patient with retinitis pigmentosa was partially recovered through stimulation with light of a specific wavelength (595 nm) formed via special goggles [101]. The human rod opsin, rhodopsin, has also been used by Kubota Vision as an optogenetics‐based gene therapy to treat retinitis pigmentosa [102]. Although rhodopsin is sensitive to light, its kinetics are too slow to support vision. In addition, multicharacteristic opsin (MCO1), an engineered microbial opsin, is being tested by Nanoscope Therapeutics for delivery via AAV‐2 into bipolar cells for retinitis pigmentosa treatment [102].

## 2 DISCUSSION AND PROSPECTIVE

By combining synthetic biology and optogenetics, multiple optogenetic circuits have been designed to induce gene expression and gene editing, activate or inhibit cell signalling and metabolic pathways, and control cell migration. The greatest advantages of optogenetic tools, specifically their non‐invasiveness, reversibility, and high spatiotemporal specificity, make up for the shortcomings of genetic switches triggered by chemical inducers [103]. In particular, phototherapy is an excellent alternative to traditional therapies for disease treatment as a non‐invasive trigger that can be rapidly removed. Therefore, optogenetic systems have been applied to gene‐ and cell‐based therapies to treat metabolic diseases, cardiovascular diseases, neurological diseases, and cancer [104].

However, clinical trials of optogenetic systems with the simple gene delivery of opsins are limited to neurodegenerative eye diseases. For clinical applications in other diseases, such as metabolic diseases and cancer, many hurdles need to be overcome, including tissue damage caused by component transplantation, phototoxicity, and limited efficiency of photocontrol. Although AAVs have been validated to deliver genes to humans, the sizes of most optogenetic systems are too large for AAVs. Several novel transplant materials and delivery methods have been developed to reduce the discomfort with gene/cell implantation; however, wounds caused by transplantation, as well as possible toxicity of transplantation carriers, can cause irreversible damage. Further, the longevity, functional durability, and potential long‐term complications of implanted cells remain to be investigated. Moreover, low tissue penetration limits human applications mostly to gene and cell therapies regulated by FRL and NIR light. In addition, the safety and stability of chassis cells are critical to realise long‐term cell therapy in translational applications. Autologous cells derived from patients, such as mesenchymal stem cells, that are immune‐compatible and non‐carcinogenic represent a good source for cell engineering. Improved encapsulation and delivery strategies may accelerate translation of optogenetic approaches to the clinic.

Multi‐light control systems designed and constructed using different wavelengths of light can be integrated to control multiple signalling pathways or multiple nodes in a pathway [105]. Further, optogenetic systems can be combined with cell and metabolite imaging and monitoring, and integrated electronically. The improvements in optical components, software programming, and electronic information technology has provided technical support for controllable light sources [106, 107]. Further improvements in optogenetic approaches as well as supportive systems are expected to ultimately lead to clinical applications.

## CONFLICT OF INTEREST

No conflicts of interest.

## PERMISSION TO REPRODUCE MATERIALS FROM OTHER SOURCES

None.

## Data Availability

Data sharing is not applicable to this article as no new data were created or analysed in this study.
